# Comparison of faecal microbiota in *Blastocystis*-positive and *Blastocystis*-negative irritable bowel syndrome patients

**DOI:** 10.1186/s40168-016-0191-0

**Published:** 2016-08-31

**Authors:** Robyn Nagel, Rebecca J. Traub, Richard J. N. Allcock, Marcella M. S. Kwan, Helle Bielefeldt-Ohmann

**Affiliations:** 1 School of Veterinary Science, The University of Queensland, Gatton Campus, Brisbane, Queensland 4343 Australia; 2 Faculty of Veterinary and Agricultural Sciences, University of Melbourne, Parkville, Melbourne, Victoria 3052 Australia; 3 School of Pathology and Laboratory Medicine, University of Western Australia, Crawley, Western Australia 6009 Australia; 4 Rural Clinical School, School of Medicine, The University of Queensland, Toowoomba, 4350 Australia; 5 Australian Infectious Diseases Research Centre, The University of Queensland, St. Lucia, Queensland 4072 Australia; 6 Toowoomba Gastroenterology Clinic, Suite 105 Medici Medical Centre, 15 Scott St, Toowoomba, QLD 4350 Australia

**Keywords:** Faecal microbiota, *Blastocystis*, Irritable bowel syndrome

## Abstract

**Background:**

We investigated whether the carriage of *Blastocystis* in IBS patients was associated with differences in the faecal microbiota. Forty patients with diarrhoea-predominant IBS (26 *Blastocystis*-positive and 14 *Blastocystis*-negative) and 57 healthy controls (HC) (42 *Blastocystis*-positive and 15 *Blastocystis*-negative) submitted faecal samples for metataxonomic analysis of the 16S ribosomal RNA gene. Differences in the relative abundance of bacteria in these IBS and HC groups were evaluated from phylum to genus level.

**Results:**

Significant changes were observed in two dominant phyla in IBS patients, regardless of *Blastocystis* infection status, namely a rise in Firmicutes and a statistically significant reduction in relative abundance of Bacteroidetes (with a threefold increase in the Firmicutes to Bacteoridetes ratio). Significant differences at genus level in IBS subjects compared to HC were also observed for many bacterial species. However, further clinical subgroup analysis of *Blastocystis*-positive and *Blastocystis-*negative subjects, regardless of symptoms, showed no significant differences at the phylum or genus level in IBS-P compared to IBS-N.

**Conclusions:**

Significant differences in the faecal microbiota between diarrhoea-predominant IBS patients and healthy controls were confirmed, but the carriage of *Blastocystis* did not significantly alter the faecal microbiota. If *Blastocystis*-positive patients represent a separate clinical subtype of IBS, this group is not identified by changes in the microbiota.

**Electronic supplementary material:**

The online version of this article (doi:10.1186/s40168-016-0191-0) contains supplementary material, which is available to authorized users.

## Background

Human newborn gut contains few organisms at birth [1] but within hours is colonised by organisms originating from the mother, diet and environment. Over 90 % of the faecal mass is microbial, the “faecal microbiota” comprises bacteria (93 %), viruses (5.8 %), archaea (0.8 %) and eukaryotes (0.5 %) [2]. Metataxonomic analysis allows insights into the relative composition of the faecal microbiota with most attention given to the bacterial component in the literature. Of the estimated 63–84 bacterial phyla described to date in humans [3], around 15 are represented in the gastrointestinal tract. Ninety percent of the colonic microbiota consists of two dominant phyla, namely Firmicutes and Bacteroidetes, with great individual variability seen at species and strain level.

The adult faecal microbiota has great inter-individual variability but is relatively stable over time in individuals [4]. Factors that influence the human faecal microbiota include mode of delivery, feeding patterns in early infancy and long-term dietary choices, immunisation, antibiotic usage, sanitation [5] and gender [6].

Although eukaryotes comprise less than 1 % of the total faecal microbiota, compositional analysis has identified 37 eukaryotic species in the faeces of healthy adults, including *Blastocystis* spp., 18 plant species and 18 fungal species [7]. A recent study of 105 healthy adults showed the prevalence of *Blastocystis* carriage to be as high as 56 %, with diverse subtypes and with stable carriage seen over a duration of 6–10 years in ten subjects [8], suggesting *Blastocystis* carriage may be one of the components of a healthy faecal microbiota.

*Blastocystis* spp., first described 100 years ago, are common, anaerobic, unicellular enteric protozoa found in almost all species of animal worldwide. The organism is found in the lumen of the terminal ileum and caecum of humans, is non-invasive and requires the presence of faecal bacterial flora for optimum growth [9]. The life cycle is still unknown although indirect and direct faecal-oral transmission likely occurs via robust cysts.

Irritable bowel syndrome (IBS) is a chronic heterogeneous condition affecting approximately 10 % of the population worldwide [10]. The disease is characterised by a clinical symptom complex and classified according to the predominant bowel habit, namely diarrhoea, constipation or “mixed” diarrhoea/constipation (IBS-D, IBS-C, IBS-M) [11].

*Blastocystis* spp. are reportedly 2.3 times more likely to be found in the stools of patients with IBS [12] and three times more likely in diarrhoea-predominant IBS patients [13] compared to healthy controls. These findings make this parasite of particular interest when investigating the faecal microbiota of patients with IBS. Although some reports link certain *Blastocystis* subtypes with increased virulence [14], no definite association has been established.

The faecal microbiota is altered in IBS and characteristically displays decreased diversity of organisms, temporal instability and changes in the phyla, particularly an increased Firmicutes to Bacteroidetes ratio [15]. Changes in the relative abundance of many other bacterial families/species in IBS are also reported [16–20]. The discordance between reported changes may be related to the particular clinical subtype of IBS or other confounding factors such as diet [21].

A previous study has suggested that irritable bowel subtypes may be characterised by their faecal microbiota profile and that these subtypes do not necessarily correspond to their clinical categorisation [20, 22]. We hypothesised that *Blastocystis* spp. are one cause of IBS, but as the individual parasites are not intrinsically pathogenic, they may produce symptoms by influencing the faecal microbiota. In this study, we compared the faecal microbiota in diarrhoea-predominant IBS patients, positive and negative for *Blastocystis* with healthy controls, positive and negative for *Blastocystis* carriage.

## Methods

### Study outline

Forty patients presenting with IBS-D to the Toowoomba Gastroenterology Clinic and 57 healthy volunteers (healthy control subjects (HC)) enrolled. Single baseline faecal samples were collected from all subjects and tested for the carriage of *Blastocystis*. The faeces were frozen at −20 °C within 4 h of collection. Extracted deoxyribonucleic acid (DNA) was stored for 6–36 months at −20 °C before being subjected to analysis for the presence/subtype of *Blastocystis* and faecal microbiota compositional analysis. Comparative analysis was made between subjects with IBS and HC and between *Blastocystis*-positive and *Blastocystis*-negative IBS (IBS-P, IBS-N) and HC (HC-P, HC-N) subjects.

### Inclusion protocol

Patients presenting to the clinic with chronic diarrhoea from 1 August 2011 to 20 February 2014 were assessed [23], including a medical consultation and examination, blood tests (full blood count, electrolytes, thyroid function tests, celiac antibodies), stool microscopy and culture and upper and lower endoscopy with duodenal biopsy for histology and disaccharidase estimation, gastric biopsy and random ileal and colonic biopsies. Forty eligible symptomatic patients, who had no other cause for symptoms identified and who fulfilled the Rome criteria for diarrhoea-predominant IBS [11], were enrolled in the study. Healthy volunteers were recruited from the University of Queensland and from asymptomatic members of households containing a symptomatic *Blastocystis*-positive patient. HC individuals were enrolled if they were in general good health with no current gastrointestinal symptoms; no past medical or medication history was taken. All patients who were invited to participate consented to enrolment and completed the study. No record was taken in any subject of diet, pre- or pro-biotic intake.

### Exclusion protocol

Non-pregnant subjects between 15 and 75 years of age were recruited for the study. Patients with significant systemic diseases or co-morbidities were excluded. Subjects were excluded if they had had a course of any antibiotic in the preceding 6 weeks prior to stool collection.

### Diagnostic methods

#### Identification of *Blastocystis*

All samples were run in parallel for the presence of *Blastocystis* spp. using an unstained wet faecal smear and xenic in vitro culture (XIVC).

DNA was extracted from faecal samples using the QIAamp DNA Stool Mini Kit (Qiagen, Hilden, Germany) according to Nagel et al. [23]. The genomic DNA from stool and faecal cultures from all subjects were subjected to polymerase chain reaction (PCR) analyses to test for the presence of *Blastocystis* [23] using the nested Wong protocol [24]. All positive PCR products were subjected to DNA sequencing and phylogenetic analysis to identify the particular ST [23]. A patient was considered to be positive for *Blastocystis* if any one of the tests was positive.

#### Compositional analysis of faecal DNA using 16S rRNA genes

The primer sequences and protocol was based on Caporaso et al. [24], with local modifications. Faecal DNA was extracted as described above and quantified using a Qubit fluorometer, and 1-ng samples were amplified using the 16S ribosomal ribonucleic acid (rRNA) gene V4/5 primers (515F: GTGCCAGCMGCCGCGGTAA and 806R: GGACTACHVGGGTWTCTAAT) (Additional file 1). Specifically, we used a mixture of gene-specific primers and gene-specific primers tagged with ion torrent-specific sequencing adaptors and barcodes. The tagged and untagged primers were mixed at a ratio of 90:10. Using this method, the approximately 10 cycle inhibition observed by using long tagged primers could be reversed, and hence, we achieved amplification of all samples using 18–20 cycles, thus minimising primer-dimer formation and allowing streamlined downstream purification. Amplification was confirmed by agarose gel electrophoresis, and product formation was quantified by fluorometry. Up to 100 amplicons were diluted to equal concentrations and adjusted to a final concentration of 15 pM. Templated Ion Shere Particles (ISP) were generated on an Ion One Touch 2 (Life Technologies) using 400 bp templating kit and sequenced on a PGM (LifeTechnologies) for 800 cycles using 400 bp sequencing kit yielding a modal read length of 309 bp. Reads were trimmed for quality purposes using TorrentSuite 4.0.2 [24]. This method has been tested on commercial mock community DNA samples and shows good concordance with expected results (data not shown).

#### Analysis of 16S rRNA gene sequences

Metataxonomic analysis using culture-independent high throughput 16SSU rRNA quantitative gene sequencing and microarrays was performed on the PCR-derived sequences. The data was analysed using software analysis programme Quantitative Insights into Microbial Ecology (QIIME, version 1.7) [24]. The following commands were applied to the derived 16S rRNA gene sequences [25]: (i) the rRNA gene sequence FASTq reads were separated into two separate libraries, one containing “sequences (FASTA files)” and the other “quality of DNA information (QUAL)” scores; (ii) each file in the sequence library was assigned a unique subject identity barcode, creating a “mapping” library; (iii) PCR “mixed sequence” chimaeras were removed using a reference file and identification of “de novo” chimeric sequences; (iv) operational taxonomic units based on 97 % specific16S rRNA gene sequence identities were used to distinguish different species of microbes, and these were grouped into their most likely phylum/class/order/family/genus using GreenGenes database, Version 12_10) [26]. Genomic analysis was obtained from taxonomic levels 1–6, but not including level 7 species subtype identification [24]. For diversity analyses, all samples were rarefied to 5000 reads per sample, and hence, all presented analyses are relative comparisons. Alpha and beta-diversity analyses were performed on the samples, and the latter was used to create Principal Coordinates Analysis (PCoA) graphs.

### Statistical analysis

Statistical analysis was carried out using IBM SPSS Statistics (IBM SPSS Statistics for Windows, Version 22.0. Armonk, NY: IBM Corp).

Percentages (relative abundance) of gut microbiota at phylum and genus level across the four clinical groups were analysed using Kruskal-Wallis test. Those species with a significant overall difference were further analysed for between group differences using the following equation [27]:

$$ \mathrm{RBari}\hbox{-} \mathrm{RBarj}\Big|>{Z}^{\ast}\mathrm{Sqrt}\left[{\left({N}^{\ast}\left(N+1\right)/12\right)}^{\ast}\left(1/\mathrm{n}\mathrm{i}+1/\mathrm{n}\mathrm{j}\right)\right] $$

where RBari, RBarj, ni and nj are the mean of the ranks and the sample sizes associated with the *i*th and *j*th groups. *N* is the total sample size, and *Z* is the critical value from the standard normal curve (*Z* = 2.638 for *k* = 4 groups and where alpha = 0.05/(k*(*k* − 1)) = 0.0083333). Statistical significance of multiple comparisons was adjusted using Bonferroni correction.

## Results

### Subjects

Table 1 shows the age, gender, medication history, *Blastocystis* status and subtype of all subjects (Table 1). A female predominance was found in the IBS group (*λ*^2^ = 15.25, *p* < 0.05).

**Table 1:** Characteristics of clinical subgroups

	IBS-P	IBS-N	HC-P	HC-N
	(*n* = 26)	(*n* = 13)	(*n* = 42)	(*n* = 13)
Age				
(mean ± sd)	45.6 ± 13.6	45.8 ± 14.0	41.8 ± 15.6	41.2 ± 13.4
Female (*n*, %)	20 (76.9)	10 (76.9)	15 (38.5)	9 (69.2)
*Blastocystis* subtypes (*n*, %)				
ST1	5 (19.2)		12 (28.6)	
ST3	8 (30.8)		12 (28.6)	
ST4	7 (26.9)		6 (14.3)	
Other subtypes (including ST2,5–8)	6 (23.1)		7 (28.6)	
Medications (*n*, %)				
Subjects on PPI/H_2_Bl	7 (27 %)	4 (29 %)		
Nil or OCP only	14 (54 %)	4 (29 %)		

*IBS-P* patients with irritable bowel syndrome positive for *Blastocystis*, *IBS-N* patients with irritable bowel syndrome negative for *Blastocystis*, *HC-P* healthy controls positive for *Blastocystis*, *HC-N* healthy controls negative for *Blastocystis*, *PPI* proton pump inhibitor therapy, *H*_*2*_ *Bl* histamine 2 blocker therapy, *OCP* oral contraceptive pill

### Bacterial phyla seen in the study subjects

Metataxonomic analysis was performed on 97 subjects (Additional file 1). The two bacterial phyla with the highest relative abundance were Firmicutes and Bacteroidetes (46.27 and 40.99 %, respectively) (Table 2). Between-gender differences of relative abundance were found in some bacterial species at the genus level (Additional file 2: Table S1), but none at the phylum level (all *p* > 0.05).

**Table 2:** Mean relative abundance of bacterial phyla seen in clinical subgroups (%)

Phyla	Total IBS	IBS-P	IBS-N	Total HC	HC-P	HC-N
	(*n* = 39)	(*n* = 26)	(*n* = 13)	(*n* = 55)	(*n* = 42)	(*n* = 13)
Actinobacteria	3.562	2.906	5.145	2.450	0.668	4.600
Bacteroidetes	34.623^a^	39.171	25.515	47.700	48.467	45.222
Cyanobacteria/chloroplast	0.032	0.045	0.006	0.023	0.025	0.015
Elusimicrobia	0.016	0.025	0	0.001	0.0005	0.003
Firmicutes	49.812	44.350	60.735	41.431	41.970	39.686
Fusobacteria	0.279	0.031	0.775	0.084	0.110	0
Lentisphaerae	0.021	0.025	0.011	0.018	0.020	0.012
Other	3.882	4.893	1.858	3.297	3.481	2.700
Proteobacteria	7.031	8.032	5.029	5.417	4.758	7.545
Spirochaetes	0	0	0	0.001	0.002	0
Synergistetes	0.007	0.004	0.012	0.003	0.003	0.005
TM7	0	0	0	0.0004	0.0005	0
Tenericutes	0	0	0	0.004	0.005	0
Verrucomicrobia	0.316	0.217	0.515	0.308	0.347	0.183
Unclassified	0.004^a^	0.006	0	0	0	0

^a^Significant difference in total IBS cf total HC using Mann-Whitney test

*IBS-P* patients with irritable bowel syndrome positive for *Blastocystis*, *IBS-N* patients with irritable bowel syndrome negative for *Blastocystis*, *HC-P* healthy controls positive for *Blastocystis*, *HC-N* healthy controls negative for *Blastocystis*, *cf* compare

### Comparison of bacterial profiles in subjects with and without IBS

Bacteroidetes relative abundance was significantly reduced in the IBS group, and the Firmicutes to Bacteroidetes ratio was three times higher in the IBS group compared to the HC (Tables 2 and 3) (*p* = 0.02).

**Table 3:** Firmicutes to Bacteroidetes ratio in clinical subgroups (abundance of Firmicutes/abundance of Bacteroidetes)

	Total IBS	IBS-P	IBS-N	Total HC	HC-P	HC-N
Firmicutes/Bacteroidetes ratio (mean ± standard deviation)	7.13 ± 13.40^a^	6.19 ± 14.85	9.00 ± 10.16	2.28 ± 7.19	1.42 ± 1.179	5.08 ± 14.51

^a^Significant difference in total IBS cf total HC using Mann-Whitney test

*IBS-P* patients with irritable bowel syndrome positive for *Blastocystis*, *IBS-N* patients with irritable bowel syndrome negative for *Blastocystis*, *HC-P* healthy controls positive for *Blastocystis*, *HC-N* healthy controls negative for *Blastocystis*, *cf* compare

A number of genera of microbes showed differences in relative abundance between IBS and HC subjects, and many of these differences reached statistical significance (Table 4).

**Table 4:** Comparison of bacterial profiles in subjects with and without IBS

Phylum (L2)	Class (L3)	Order (L4)	Family (L5)	Genus (L6)
Euryarchaeota	***Methanobacteria*** *↑*	***Methanobacteriales*** *↑*	***Methanobacteriaceae*** *↑*	***Methanobrevibacter*** *↑*
Actinobacteria	*Actinobacteria*	***Actinomycetales*** *↑*	***Actinomycetaceae*** *↑*	***Actinomyces*** *↑*
		*Bifidobacteriales*	*Bifidobacteriaceae*	***Other*** *↑*
		*Coriobacteriales*	*Coriobacteriaceae*	***Eggethella*** *↑*
				***Gordonibacter*** *↑*
				***Olsenella*** *↓*
***Bacteroidetes*** *↓*	***Bacteroidia*** *↓*	***Bacteroidales*** *↓*	*Porphyromonadaceae*	***Butyricimonas*** *↓*
				***Parabacteroides*** *↓*
Firmicutes	*Bacilli*	*Lactobacillales*	*Enterococcaceae*	***Enterococcus*** *↑*
			***Streptococcaceae*** *↑*	***Streptococcus*** *↑*
	***Clostridia*** *↑*	***Clostridiales*** *↑*	***Lachnospiraceae*** *↑*	***Anaerostipes*** *↑*
				***Blautia*** *↑*
				***Lachnospiracea_incertae_sedis*** *↑*
			***Peptococcaceae 1*** *↓*	***Peptococcus*** *↓*
			*Rumincoccaceae*	***Papillibacter*** *↑*
	*Erysipelotricha*	*Erysipelotrichales*	*Erysipelotrichaceae*	***Cantenibacterium*** *↓*
				***Other*** *↑*
	***Negativicutes*** *↓*	***Selenomonadales*** *↓*	***Veillonellaceae*** *↓*	***Allisonella*** *↓*
				***Dialister*** *↓*
Proteobacteria	***Alphaproteobacteria*** *↑*	***Rhizobiales*** *↑*	***Hyphomicrobiaceae*** *↑*	***Gemmiger*** *↑*
***(Unclassified)*** *↑*	***Other*** *↑*	***Other*** *↑*	***Other*** *↑*	***Other*** *↑*

Bold entries indicate significant difference between groups (*p* < 0.05). ↑ and ↓ indicate significant (*p* < 0.05) increase or decrease in IBS relative to healthy subjects, respectively

*L* level

### Comparison of bacterial profiles across the four clinical subgroups

No significant differences were found between major bacterial phyla profiles in IBS-P and IBS-N patients (Table 2). The minor phyla only have small numbers of subjects in each group making meaningful statistical interpretation difficult.

**Fig. 1:**
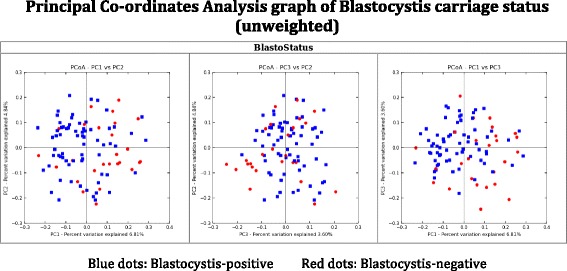
Title: Principal Co-ordinates Analysis graph of *Blastocystis* carriage status (unweighted). Legend: *Blue dots Blastocystis*-positive, *red dots Blastocystis*-negative

**Fig. 2:**
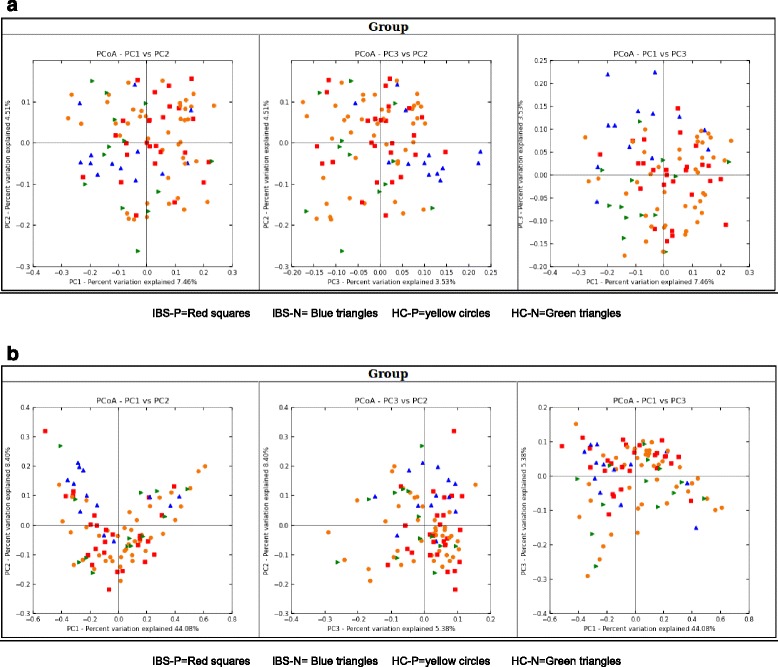
**a** Title: Principal Co-ordinates Analysis graph of all clinical subgroups (unweighted). Legend: *IBS-P* red squares, *IBS-N* blue triangles, *HC-P* yellow circles, *HC-N* green triangles. **b** Title: Principal Coordinates Analysis graph of all clinical subgroups (weighted). Legend: *IBS-P* red squares, *IBS-N* blue triangles, *HC-P* yellow circles, *HC-N* green triangles

Significant differences in bacterial profiles at genus level were not found between the clinical subgroups, particularly between IBS-P and IBS-N groups (Table 5). Figure 1 (stratified for *Blastocystis* carriage), Fig. 2a (unweighted, recording presence but not accounting for abundance of different phyla and species) and Fig. 2b (weighted for differences in abundance of phyla and species) illustrate the similarities of the bacterial profile amongst the four clinical groups, with considerable overlap, and no single group found to be an outlier.

**Table 5:** Comparison of relative abundance of selected (selection based on overall statistical significance across all clinical groups, determined by Kruskal-Wallis test with *p* < 0.05) bacterial species across the four clinical subgroups

Species	Mean ± SD (%)			
	IBS-P	IBS-N	HC-P	HC-N
	(*n* = 26)	(*n* = 13)	(*n* = 42)	(*n* = 13)
Actinomyces spp.^a^	0.019 ± 0.052	0.035 ± 0.043	0.001 ± 0.005	0.015 ± 0.023
Anaerostipes spp.^ba^	0.248 ± 0.498	2.040 ± 2.235	0.123 ± 0.157	0.395 ± 0.793
Papillibacter spp.	0	0.025 ± 0.067	0	0
Blautia *spp*.^ba^	1.130 ± 1.923	6.505 ± 5.909	0.450 ± 0.366	1.975 ± 4.392
Lauconostoc spp.^ac^	0.035 ± 0.058	0.002 ± 0.006	0.063 ± 0.101	0.005 ± 0.012
Eggerthella spp.^a^	0.018 ± 0.058	0.112 ± 0.189	0.003 ± 0.010	0.008 ± 0.015
Weissella spp.	0.038 ± 0.066	0.003 ± 0.011	0.090 ± 0.172	0.002 ± 0.006
Bifidobacterium spp.^ba^	2.440 ± 8.130	3.915 ± 5.362	0.408 ± 1.039	4.195 ± 9.828
Allisonella spp.	0.008 ± 0.028	0	0.016 ± 0.049	0.149 ± 0.443
Bifidobacteriaceae, Other spp.	0.038 ± 0.156	0.025 ± 0.032	0.003 ± 0.013	0.085 ± 0.254
Streptococcus spp.^a^	1.276 ± 2.612	0.643 ± 0.657	0.192 ± 0.273	0.595 ± 1.476
Lachnospiracea_incertae_sedis spp.^a^	2.092 ± 3.592	2.600 ± 1.666	0.813 ± 0.769	1.206 ± 1.048
p_Bacteria, Other spp.^bd^	4.893 ± 5.681	1.858 ± 2.906	3.481 ± 3.747	2.700 ± 6.159
Clostridium XI spp.^a^	0.224 ± 0.378	0.911 ± 0.761	0.255 ± 0.505	0.312 ± 0.511
Eubacterium spp.	0.004 ± 0.013	0.046 ± 0.105	0.004 ± 0.013	0.015 ± 0.038
Acinetobacter spp.	0.017 ± 0.036	0.002 ± 0.006	0.031 ± 0.106	0
Dialister spp.^a^	0.227 ± 0.727	1.997 ± 4.464	3.799 ± 11.633	4.292 ± 6.127
Gordonibacter spp.	0.005 ± 0.012	0.012 ± 0.029	0	0.002 ± 0.006
Canternibacter spp.	0.004 ± 0.016	0	0.154 ± 0.546	0.046 ± 0.126
Oxalobacteraceae, Other spp.	0.049 ± 0.080	0.006 ± 0.017	0.050 ± 0.095	0.022 ± 0.072
Olsenella spp.	0	0	0.006 ± 0.023	0
Alistipes spp.^b^	6.142 ± 5.326	1.975 ± 2.507	5.473 ± 5.802	4.992 ± 5.512
Clostridium IV spp.^c^	0.829 ± 1.501	0.495 ± 0.438	1.037 ± 1.373	0.277 ± 0.400

^a^Significant post hoc difference IBS-N vs HC-P

^b^Significant post hoc difference IBS-P vs IBS-N

^c^Significant post hoc difference HC-P vs HC-N

^d^Significant post hoc difference IBS-P vs HC-N

## Discussion

Previously reported changes in the faecal microbiota of the two dominant phyla, with a raised Firmicutes to Bacteroides ratio in IBS patients compared to HC, were confirmed in this study comprising diarrhoea-predominant IBS patients. Reductions in relative abundance in our study of diarrhoea-predominant patients were in accord with *Parabacteroides* spp., but results found in our study for *Actinomyces*, *Bifidobacteriacea_Other*, *Dialister*, *Veillonellaceae* and *Methanobrevibacter* spp. differed from previous results reported for constipation-predominant IBS [16].

Many studies of the faecal microbiota in IBS patients have not separated out clinical subtypes of IBS (diarrhoea, constipation, or mixed-predominant) or other confounding factors [19], and this may account for differences in results. In our study, almost half the IBS patients were taking either no medication or only the oral contraceptive pill (OCP) and numbers of patients on medication were high in both IBS-P and IBS-N groups. Universally, IBS has a female predominance [10]. Sex hormone modulation of the gut microbiota has been reported [28], and it is likely OCP therapy has some impact on the faecal microbiota. Nevertheless, this study did not identify any changes in relative composition of phyla (and minimal changes in the genus) associated with gender (Additional file 3). Approximately one third of subjects with IBS were taking acid suppression therapy that has been reported to change the gastric microbiota significantly but have much less effect on the colonic microbiota [29].

In our study of diarrhoea-predominant IBS patients, no significant differences between the IBS-P, IBS-N, HC-P and HC-N groups were seen at the phyla or genus level. Although IBS patients have a different faecal microbiota profile compared to healthy subjects, the carriage of *Blastocystis* does not make a significant difference to this profile. This suggests that if *Blastocystis* spp. do cause some cases of IBS, mechanisms other than alteration of the faecal microbiota must be involved. It is possible that some *Blastocystis* organisms have unique, as yet undefined, pathological attributes [30] or that the host immune response may be an important factor in determining clinical response to *Blastocystis* infection [31].

## Conclusions

Changes in the faecal microbiota in the dominant phyla and the Firmicutes to Bacteroidetes ratio are confirmed in diarrhoea-predominant IBS patients compared to HC subjects. Although IBS patients with *Blastocystis* may constitute a separate clinical IBS group, this group is not characterised by changes in the faecal microbiota.

## Additional files

Additional file 1: File S1. Metaxanomic analysis of faecal samples. (DOCX 46 kb) Additional file 2: Table S1. Relative abundance (percentage) of bacterial groups (by sex). (DOCX 144 kb) Additional file 3: Clinical subgroup phyla stratified for gender. (DOCX 93 kb)

## Abbreviations

DNA: Deoxyribonucleic acid
HC: Healthy control subjects
HC-N: Healthy control subjects negative for *Blastocystis* carriage
HC-P: Healthy control subjects positive for *Blastocystis* carriage
IBS: Irritable bowel syndrome
IBS-C: Constipation-predominant IBS
IBS-D: Diarrhoea-predominant IBS
IBS-M: Mixed bowel habit IBS
IBS-N: Irritable bowel syndrome patients negative for *Blastocystis* carriage
IBS-P: Irritable bowel syndrome patients positive for *Blastocystis* carriage
PCoA: Principal Coordinates Analysis
PCR: Polymerase chain reaction
rRNA: Ribosomal ribonucleic acid
XIVC: Xenic in vitro culture

## References

[1] Jimenez E, Fernandez L, Marin ML, Martin R, Odriozola JM, Nueno-Palop C (2005). Isolation of commensal bacteria from umbilical cord blood of healthy neonates born by cesarean section. Curr Microbiol.

[2] Arumugam M, Raes J, Pelletier E, Le Paslier D, Yamada T, Mende DR (2011). Enterotypes of the human gut microbiome. Nature.

[3] Kantor RS, Wrighton KC, Handley KM, Sharon I, Hug LA, Castelle CJ (2013). Small genomes and sparse metabolisms of sediment-associated bacteria from four candidate phyla. MBio.

[4] Lozupone CA, Stombaugh JI, Gordon JI, Jansson JK, Knight R (2012). Diversity, stability and resilience of the human gut microbiota. Nature.

[5] Arrieta MC, Stiemsma LT, Amenyogbe N, Brown EM, Finlay B (2014). The intestinal microbiome in early life: health and disease. Front Immunol.

[6] Aguirre de Carcer D, Cuiv PO, Wang T, Kang S, Worthley D, Whitehall V (2011). Numerical ecology validates a biogeographical distribution and gender-based effect on mucosa-associated bacteria along the human colon. ISME J.

[7] Gouba N, Raoult D, Drancourt M (2013). Plant and fungal diversity in gut microbiota as revealed by molecular and culture investigations. PLoS One.

[8] Scanlan PD, Stensvold CR, Rajilic-Stojanovic M, Heilig HG, De Vos WM, O'Toole PW (2014). The microbial eukaryote Blastocystis is a prevalent and diverse member of the healthy human gut microbiota. FEMS Microbiol Ecol.

[9] Zierdt CH (1991). Blastocystis hominis—past and future. Clin Microbiol Rev.

[10] Canavan C, West J, Card T (2014). The epidemiology of irritable bowel syndrome. Clin epidemiol.

[11] Longstreth GF, Thompson WG, Chey WD, Houghton LA, Mearin F, Spiller RC (2006). Functional bowel disorders. Gastroenterology.

[12] Nourrisson C, Scanzi J, Pereira B, NkoudMongo C, Wawryzyniak I, Cian A (2014). Blastocystis is associated with decrease of fecal microbiota protective bacteria: comparative analysis between patients with irritable bowel syndrome and control subjects. PLoS One.

[13] Yakoob J, Jafri W, Beg MA, Abbas Z, Naz S, Islam M (2010). Irritable bowel syndrome: is it associated with genotypes of Blastocystis hominis. Parasitol Res.

[14] Hussein EM, Hussein AM, Eida MM, Atwa MM (2008). Pathophysiological variability of different genotypes of human Blastocystis hominis Egyptian isolates in experimentally infected rats. Parasitol Res.

[15] Simren M, Barbara G, Flint HJ, Spiegel BM, Spiller RC, Vanner S (2013). Intestinal microbiota in functional bowel disorders: a Rome foundation report. Gut.

[16] Bye W, Ishaq N, Bolin TD, Duncombe VM, Riordan SM (2014). Overgrowth of the indigenous gut microbiome and irritable bowel syndrome. World J Gastroenterol.

[17] Rajilić–Stojanović M, Biagi E, Heilig HGHJ, Kajander K, Kekkonen RA, Tims S (2011). Global and deep molecular analysis of microbiota signatures in fecal samples from patients with irritable bowel syndrome. Gastroenterology.

[18] Malinen E, Rinttila T, Kajander K, Matto J, Kassinen A, Krogius L (2005). Analysis of the fecal microbiota of irritable bowel syndrome patients and healthy controls with real-time PCR. Am J Gastroenterol.

[19] Lyra A, Rinttilä T, Nikkilä J, Krogius-Kurikka L, Kajander K, Malinen E (2009). Diarrhoea-predominant irritable bowel syndrome distinguishable by 16S rRNA gene phylotype quantification. World J Gastroenterol.

[20] Jeffery IB, O'Toole PW, Ohman L, Claesson MJ, Deane J, Quigley EM (2012). An irritable bowel syndrome subtype defined by species-specific alterations in faecal microbiota. Gut.

[21] Rajilic-Stojanovic M, Jonkers DM, Salonen A, Hanevik K, Raes J, Jalanka J (2015). Intestinal microbiota and diet in IBS: causes, consequences, or epiphenomena?. Am J Gastroenterol.

[22] Jalanka-Tuovinen J, Salojarvi J, Salonen A, Immonen O, Garsed K, Kelly FM (2014). Faecal microbiota composition and host-microbe cross-talk following gastroenteritis and in postinfectious irritable bowel syndrome. Gut.

[23] Nagel R, Bielefeldt-Ohmann H, Traub RJ (2014). Clinical pilot study: efficacy of triple antibiotic therapy in Blastocystis positive irritable bowel syndrome patients. Gut Pathogens.

[24] Caporaso JG, Kuczynski J, Stombaugh J, Bittinger K, Bushman FD, Costello EK (2010). QIIME allows analysis of high-throughput community sequencing data. Nat Methods.

[25] Cock PJ, Fields CJ, Goto N, Heuer ML, Rice PM (2010). The Sanger FASTQ file format for sequences with quality scores, and the Solexa/Illumina FASTQ variants. Nucleic Acids Res.

[26] McDonald D, Price MN, Goodrich J, Nawrocki EP, DeSantis TZ, Probst A (2012). An improved Greengenes taxonomy with explicit ranks for ecological and evolutionary analyses of bacteria and archaea. ISME J.

[27] Siegel S, Castellan NJ (1988). Nonparametric statistics for the behavioral sciences.

[28] Mulak A, Tache Y, Larauche M (2014). Sex hormones in the modulation of irritable bowel syndrome. World J Gastroenterol.

[29] Tsuda A, Suda W, Morita H, Takanashi K, Takagi A, Koga Y (2015). Influence of proton-pump inhibitors on the luminal microbiota in the gastrointestinal tract. Clin trans gastroenterol.

[30] Poirier P, Wawrzyniak I, Vivares CP, Delbac F, El Alaoui H (2012). New insights into Blastocystis species; a potential link with irritable bowel syndrome. PLoS Pathog.

[31] Nagel R, Traub RJ, Kwan M, Bielefeldt-Ohmann H (2015). Blastocystis specific serum immunoglobulin in patients with irritable bowel sydrome versus healthy controls. Parasites Vectors.

