# Supplementary material for: Comparison of faecal microbiota in Blastocystis-positive and Blastocystis-negative irritable bowel syndrome patients
Source: Microbiome. 2016 Aug 31;4(1):47. doi: 10.1186/s40168-016-0191-0 (PMC5007835; doi:10.1186/s40168-016-0191-0)
Supplement: Additional file 1: File S1. — Metaxanomic analysis of faecal samples. (DOCX 46 kb) [file 40168_2016_191_MOESM1_ESM.docx]

File s1

**Metataxonomic Analysis protocol**

We performed 16S amplification and sequencing analysis on DNA samples derived from the 97 patients. We produced an average of 20813 (range 10-60302) reads per sample. Three patients did not generate enough microbiological DNA sequences and the statistical analysis was performed on a total of 39 IBS patients (26 positive, 13 negative) and 55 healthy controls (42 positive, 13 negative). Within the included samples, we observed an average of 302 OTUs per sample (range 127-521)
