# Supplementary material for: Comparison of faecal microbiota in Blastocystis-positive and Blastocystis-negative irritable bowel syndrome patients
Source: Microbiome. 2016 Aug 31;4(1):47. doi: 10.1186/s40168-016-0191-0 (PMC5007835; doi:10.1186/s40168-016-0191-0)
Supplement: Additional file 2: Table S1. — Relative abundance (percentage) of bacterial groups (by sex). (DOCX 144 kb) [file 40168_2016_191_MOESM2_ESM.docx]

**Table s1. Relative abundance (percentage) of bacterial groups (by sex).**

| Group / *Blastocystis* qPCR result | Subjects | *Actinomyces* sp. | | | *Bifidobacterium* sp. | | | *Clostridium senso stricto* sp. | | | *Bilophila* sp. | | |
| --- | --- | --- | --- | --- | --- | --- | --- | --- | --- | --- | --- | --- | --- |
|  |  | Mean | SD | *p*-value | Mean | SD | *p*-value | Mean | SD | *p*-value | Mean | SD | *p*-value |
| IBS-N | All | 0.035 | 0.043 | >0.05 | 3.915 | 5.362 | >0.05 | 0.495 | 0.729 | >0.05 | 0.106 | 0.194 | >0.05 |
| HC-N |  | 0.015 | 0.023 |  | 4.195 | 9.828 |  | 0.334 | 0.707 |  | 0.286 | 0.314 |  |
| IBS-N | Females | 0.036 | 0.048 | >0.05 | 2.688 | 2.775 | >0.05 | 0.614 | 0.797 | **<0.05** | 0.138 | 0.213 | >0.05 |
| HC-N |  | 0.020 | 0.026 |  | 5.764 | 11.637 |  | 0.178 | 0.440 |  | 0.358 | 0.335 |  |
| IBS-N | Males | 0.033 | 0.023 | >0.05 | 8.007 | 10.257 | >0.05 | 0.100 | 0.173 | >0.05 | 0 | 0 | >0.05 |
| HC-N |  | 0.005 | 0.010 |  | 0.665 | 1.131 |  | 0.685 | 1.117 |  | 0.125 | 0.212 |  |
| IBS-N | All | 0.035 | 0.043 | >0.05 | 3.915 | 5.362 | **<0.05** | 0.495 | 0.729 | >0.05 | 0.106 | 0.194 | >0.05 |
| IBS-P |  | 0.019 | 0.052 |  | 2.440 | 8.130 |  | 0.182 | 0.423 |  | 0.357 | 0.493 |  |
| IBS-N | Females | 0.036 | 0.048 | >0.05 | 2.688 | 2.775 | >0.05 | 0.614 | 0.797 | >0.05 | 0.138 | 0.213 | >0.05 |
| IBS-P |  | 0.023 | 0.058 |  | 3.129 | 9.208 |  | 0.193 | 0.473 |  | 0.359 | 0.517 |  |
| IBS-N | Males | 0.033 | 0.023 | >0.05 | 8.007 | 10.257 | >0.05 | 0.100 | 0.173 | >0.05 | 0 | 0 | **<0.05** |
| IBS-P |  | 0.007 | 0.010 |  | 0.143 | 0.216 |  | 0.143 | 0.206 |  | 0.350 | 0.446 |  |
| HC-P | All | 0.001 | 0.005 | >0.05 | 0.408 | 1.038 | >0.05 | 0.313 | 0.704 | >0.05 | 0.176 | 0.322 | >0.05 |
| HC-N |  | 0.015 | 0.023 |  | 4.195 | 9.828 |  | 0.334 | 0.707 |  | 0.286 | 0.314 |  |
| HC-P | Females | 0.001 | 0.005 | >0.05 | 0.208 | 0.413 | >0.05 | 0.147 | 0.287 | >0.05 | 0.304 | 0.482 | >0.05 |
| HC-N |  | 0.020 | 0.026 |  | 5.764 | 11.637 |  | 0.178 | 0.440 |  | 0.358 | 0.335 |  |
| HC-P | Males | 0.002 | 0.006 | >0.05 | 0.574 | 1.322 | >0.05 | 0.414 | 0.892 | >0.05 | 0.110 | 0.160 | >0.05 |
| HC-N |  | 0.005 | 0.010 |  | 0.665 | 1.131 |  | 0.685 | 1.117 |  | 0.125 | 0.212 |  |

| Group / *Blastocystis* qPCR result | Subjects | *Dialister* sp. | | | *Parabacteroides* sp. | | | *Alistipes* sp. | | | *Leuconostoc* sp. | | |
| --- | --- | --- | --- | --- | --- | --- | --- | --- | --- | --- | --- | --- | --- |
|  |  | Mean | SD | *p*-value | Mean | SD | *p*-value | Mean | SD | *p*-value | Mean | SD | *p*-value |
| IBS-N | All | 1.997 | 4.464 | >0.05 | 1.800 | 2.333 | >0.05 | 1.975 | 2.507 | >0.05 | 0.002 | 0.006 | >0.05 |
| HC-N |  | 4.292 | 6.127 |  | 3.954 | 4.320 |  | 4.992 | 5.512 |  | 0.005 | 0.012 |  |
| IBS-N | Females | 2.596 | 4.985 | >0.05 | 1.414 | 1.745 | >0.05 | 2.328 | 2.750 | >0.05 | 0 | 0 | >0.05 |
| HC-N |  | 3.607 | 4.954 |  | 4.862 | 4.967 |  | 6.140 | 6.143 |  | 0.002 | 0.007 |  |
| IBS-N | Males | 0 | 0 | >0.05 | 3.087 | 3.947 | >0.05 | 0.800 | 0.999 | >0.05 | 0.007 | 0.012 | >0.05 |
| HC-N |  | 5.835 | 8.951 |  | 1.910 | 0.903 |  | 2.410 | 2.839 |  | 0.010 | 0.020 |  |
| IBS-N | All | 1.997 | 4.464 | >0.05 | 1.800 | 2.333 | >0.05 | 1.975 | 2.507 | **<0.05** | 0.002 | 0.006 | >0.05 |
| IBS-P |  | 0.227 | 0.727 |  | 2.586 | 2.403 |  | 6.142 | 5.326 |  | 0.035 | 0.058 |  |
| IBS-N | Females | 2.596 | 4.985 | >0.05 | 1.414 | 1.745 | >0.05 | 2.328 | 2.750 | >0.05 | 0 | 0 | >0.05 |
| IBS-P |  | 0.295 | 0.822 |  | 2.194 | 2.325 |  | 5.912 | 5.684 |  | 0.039 | 0.062 |  |
| IBS-N | Males | 0 | 0 | >0.05 | 3.087 | 3.947 | >0.05 | 0.800 | 0.999 | >0.05 | 0.007 | 0.012 | >0.05 |
| IBS-P |  | 0 | 0 |  | 3.893 | 2.383 |  | 6.907 | 4.260 |  | 0.020 | 0.040 |  |
| HC-P | All | 3.799 | 11.633 | >0.05 | 3.273 | 3.001 | >0.05 | 5.473 | 5.802 | >0.05 | 0.063 | 0.101 | **<0.05** |
| HC-N |  | 4.292 | 6.127 |  | 3.954 | 4.320 |  | 4.992 | 5.512 |  | 0.005 | 0.012 |  |
| HC-P | Females | 0.880 | 2.118 | >0.05 | 4.709 | 3.984 | >0.05 | 7.536 | 5.636 | >0.05 | 0.104 | 0.121 | **<0.05** |
| HC-N |  | 3.607 | 4.954 |  | 4.862 | 4.967 |  | 6.140 | 6.143 |  | 0.002 | 0.007 |  |
| HC-P | Males | 5.934 | 15.064 | >0.05 | 2.592 | 2.000 | >0.05 | 4.808 | 5.846 | >0.05 | 0.046 | 0.086 | >0.05 |
| HC-N |  | 5.835 | 8.951 |  | 1.910 | 0.903 |  | 2.410 | 2.839 |  | 0.010 | 0.020 |  |

| Group / *Blastocystis* qPCR result | Subjects | *Weisseila* sp. | | | *Anaerostipes* sp. | | | *Blautia* sp. | | | | *Clostridium XI* sp. | | |
| --- | --- | --- | --- | --- | --- | --- | --- | --- | --- | --- | --- | --- | --- | --- |
|  |  | Mean | SD | *p*-value | Mean | SD | *p*-value | Mean | SD | *p*-value | Mean | | SD | *p*-value |
| IBS-N | All | 0.003 | 0.011 | >0.05 | 2.040 | 2.235 | >0.05 | 6.505 | 5.909 | **<0.05** | 0.911 | | 0.761 | >0.05 |
| HC-N |  | 0.002 | 0.006 |  | 0.395 | 0.793 |  | 1.975 | 4.392 |  | 0.312 | | 0.511 |  |
| IBS-N | Females | 0.004 | 0.013 | >0.05 | 2.174 | 2.515 | >0.05 | 5.782 | 6.138 | >0.05 | 0.850 | | 0.745 | >0.05 |
| HC-N |  | 0 | 0 |  | 0.538 | 0.930 |  | 2.267 | 5.139 |  | 0.244 | | 0.538 |  |
| IBS-N | Males | 0 | 0 | >0.05 | 1.593 | 1.051 | **<0.05** | 8.913 | 5.350 | **<0.05** | 1.113 | | 0.948 | >0.05 |
| HC-N |  | 0.005 | 0.010 |  | 0.075 | 0.111 |  | 1.320 | 2.428 |  | 0.465 | | 0.476 |  |
| IBS-N | All | 0.003 | 0.011 | >0.05 | 2.040 | 2.235 | **<0.05** | 6.505 | 5.909 | **<0.05** | 0.911 | | 0.761 | >0.05 |
| IBS-P |  | 0.038 | 0.066 |  | 0.248 | 0.498 |  | 1.130 | 1.923 |  | 0.224 | | 0.378 |  |
| IBS-N | Females | 0.004 | 0.013 | >0.05 | 2.174 | 2.515 | **<0.05** | 5.782 | 6.138 | **<0.05** | 0.850 | | 0.745 | >0.05 |
| IBS-P |  | 0.041 | 0.071 |  | 0.299 | 0.560 |  | 1.335 | 2.156 |  | 0.223 | | 0.396 |  |
| IBS-N | Males | 0 | 0 | >0.05 | 1.593 | 1.051 | >0.05 | 8.913 | 5.350 | >0.05 | 1.113 | | 0.948 | >0.05 |
| IBS-P |  | 0.030 | 0.047 |  | 0.077 | 0.051 |  | 0.447 | 0.317 |  | 0.227 | | 0.345 |  |
| HC-P | All | 0.090 | 0.172 | >0.05 | 0.123 | 0.157 | >0.05 | 0.450 | 0.366 | >0.05 | 0.255 | | 0.505 | >0.05 |
| HC-N |  | 0.002 | 0.006 |  | 0.395 | 0.793 |  | 1.975 | 4.392 |  | 0.312 | | 0.511 |  |
| HC-P | Females | 0.136 | 0.197 | **<0.05** | 0.096 | 0.113 | >0.05 | 0.452 | 0.346 | >0.05 | 0.245 | | 0.595 | >0.05 |
| HC-N |  | 0 | 0 |  | 0.538 | 0.930 |  | 2.267 | 5.139 |  | 0.244 | | 0.538 |  |
| HC-P | Males | 0.072 | 0.162 | >0.05 | 0.150 | 0.184 | >0.05 | 0.462 | 0.401 | >0.05 | 0.260 | | 0.478 | >0.05 |
| HC-N |  | 0.005 | 0.010 |  | 0.075 | 0.111 |  | 1.320 | 2.428 |  | 0.465 | | 0.476 |  |

| Group / *Blastocystis* qPCR result | Subjects | *p_Bacteria, other* sp. | | | *Streptococcus* sp. | | | *Lachnnnospiraceae incertae sedis* sp. | | | *Gemmiger* sp. | | |
| --- | --- | --- | --- | --- | --- | --- | --- | --- | --- | --- | --- | --- | --- |
|  |  | Mean | SD | *p*-value | Mean | SD | *p*-value | Mean | SD | *p*-value | Mean | SD | *p*-value |
| IBS-N | All | 1.858 | 2.906 | >0.05 | 0.643 | 0.657 | >0.05 | 2.600 | 1.666 | >0.05 | 1.868 | 2.203 | >0.05 |
| HC-N |  | 2.700 | 6.159 |  | 0.483 | 0.622 |  | 1.206 | 1.048 |  | 0.562 | 0.825 |  |
| IBS-N | Females | 2.074 | 3.234 | >0.05 | 0.676 | 0.724 | >0.05 | 2.624 | 1.691 | >0.05 | 1.312 | 1.463 | >0.05 |
| HC-N |  | 1.136 | 1.831 |  | 0.556 | 0.718 |  | 1.427 | 1.071 |  | 0.249 | 0.223 |  |
| IBS-N | Males | 1.140 | 1.613 | **<0.05** | 0.533 | 0.454 | >0.05 | 2.520 | 1.941 | >0.05 | 3.720 | 3.577 | >0.05 |
| HC-N |  | 6.220 | 10.905 |  | 0.320 | 0.348 |  | 0.710 | 0.926 |  | 1.265 | 1.280 |  |
| IBS-N | All | 1.858 | 2.906 | >0.05 | 0.643 | 0.657 | >0.05 | 2.600 | 1.666 | >0.05 | 1.868 | 2.203 | >0.05 |
| IBS-P |  | 4.893 | 5.681 |  | 1.276 | 2.612 |  | 2.092 | 3.592 |  | 2.728 | 7.328 |  |
| IBS-N | Females | 2.074 | 3.234 | >0.05 | 0.676 | 0.724 | >0.05 | 2.624 | 1.691 | >0.05 | 1.312 | 1.463 | >0.05 |
| IBS-P |  | 3.525 | 4.444 |  | 1.597 | 2.912 |  | 2.216 | 4.098 |  | 3.340 | 8.287 |  |
| IBS-N | Males | 1.140 | 1.613 | >0.05 | 0.533 | 0.454 | >0.05 | 2.520 | 1.941 | >0.05 | 3.720 | 3.577 | >0.05 |
| IBS-P |  | 9.453 | 7.339 |  | 0.207 | 0.346 |  | 1.677 | 0.655 |  | 0.687 | 0.994 |  |
| HC-P | All | 3.481 | 3.747 | >0.05 | 0.192 | 0.273 | >0.05 | 0.813 | 0.769 | >0.05 | 0.571 | 0.668 | >0.05 |
| HC-N |  | 2.700 | 6.159 |  | 0.483 | 0.622 |  | 1.206 | 1.048 |  | 0.562 | 0.825 |  |
| HC-P | Females | 3.996 | 2.797 | >0.05 | 0.277 | 0.361 | >0.05 | 0.955 | 0.777 | >0.05 | 0.615 | 0.648 | >0.05 |
| HC-N |  | 1.136 | 1.831 |  | 0.556 | 0.718 |  | 1.427 | 1.071 |  | 0.249 | 0.223 |  |
| HC-P | Males | 3.327 | 4.379 | **<0.05** | 0.158 | 0.211 | >0.05 | 0.800 | 0.791 | >0.05 | 0.467 | 0.638 | >0.05 |
| HC-N |  | 6.220 | 10.905 |  | 0.320 | 0.348 |  | 0.710 | 0.926 |  | 1.265 | 1.280 |  |

| Group / *Blastocystis* qPCR result | Subjects | *Clostridium IV* sp. | | |  | | | p_Actinobacteria | | | p_Bacteroidetes | | |
| --- | --- | --- | --- | --- | --- | --- | --- | --- | --- | --- | --- | --- | --- |
|  |  | Mean | SD | *p*-value | Mean | SD | *p*-value | Mean | SD | *p*-value | Mean | SD | *p*-value |
| IBS-N | All | 0.495 | 0.438 | >0.05 |  |  |  | 5.145 | 5.888 | >0.05 | 25.528 | 25.515 | >0.05 |
| HC-N |  | 0.277 | 0.400 |  |  |  |  | 4.600 | 10.218 |  | 45.222 | 21.909 |  |
| IBS-N | Females | 0.568 | 0.479 | >0.05 |  |  |  | 3.782 | 3.823 | >0.05 | 28.090 | 28.197 | >0.05 |
| HC-N |  | 0.362 | 0.458 |  |  |  |  | 6.069 | 12.129 |  | 49.649 | 22.175 |  |
| IBS-N | Males | 0.253 | 0.083 | >0.05 |  |  |  | 9.687 | 10.099 | >0.05 | 16.987 | 13.640 | >0.05 |
| HC-N |  | 0.085 | 0.101 |  |  |  |  | 1.295 | 2.086 |  | 35.260 | 20.435 |  |
| IBS-N | All | 0.495 | 0.438 | >0.05 |  |  |  | 5.145 | 5.888 | **<0.05** | 25.528 | 25.515 | >0.05 |
| IBS-P |  | 0.829 | 1.501 |  |  |  |  | 2.906 | 8.326 |  | 39.171 | 22.258 |  |
| IBS-N | Females | 0.568 | 0.479 | >0.05 |  |  |  | 3.782 | 3.823 | >0.05 | 28.090 | 28.197 | >0.05 |
| IBS-P |  | 0.627 | 0.858 |  |  |  |  | 3.649 | 9.415 |  | 37.508 | 23.447 |  |
| IBS-N | Males | 0.253 | 0.083 | >0.05 |  |  |  | 9.687 | 10.099 | >0.05 | 16.987 | 13.640 | >0.05 |
| IBS-P |  | 1.503 | 2.785 |  |  |  |  | 0.430 | 0.400 |  | 44.713 | 18.442 |  |
| HC-P | All | 1.037 | 1.373 | **<0.05** |  |  |  | 0.668 | 1.315 | >0.05 | 48.467 | 20.257 | >0.05 |
| HC-N |  | 0.277 | 0.400 |  |  |  |  | 4.600 | 10.218 |  | 45.222 | 21.909 |  |
| HC-P | Females | 1.208 | 1.350 | >0.05 |  |  |  | 0.535 | 0.779 | >0.05 | 48.763 | 17.747 | >0.05 |
| HC-N |  | 0.362 | 0.458 |  |  |  |  | 6.069 | 12.129 |  | 49.649 | 22.175 |  |
| HC-P | Males | 0.791 | 0.945 | >0.05 |  |  |  | 0.814 | 1.627 | >0.05 | 49.683 | 22.739 | >0.05 |
| HC-N |  | 0.085 | 0.101 |  |  |  |  | 1.295 | 2.086 |  | 35.260 | 20.435 |  |

IBS-P: patients with irritable bowel syndrome (IBS) positive for *Blastocystis*; IBS-N: patients with IBS negative for *Blastocystis*; HC-P: Healthy controls positive for *Blastocystis*; HC-N: Health controls negative for *Blastocystis*; SD: standard deviation
