# Supplementary figures and images for: Comparison of faecal microbiota in Blastocystis-positive and Blastocystis-negative irritable bowel syndrome patients
Source: Microbiome. 2016 Aug 31;4(1):47. doi: 10.1186/s40168-016-0191-0 (PMC5007835; doi:10.1186/s40168-016-0191-0)

**Supplementary Figure s1 Clinical subgroup phyla stratified for gender**


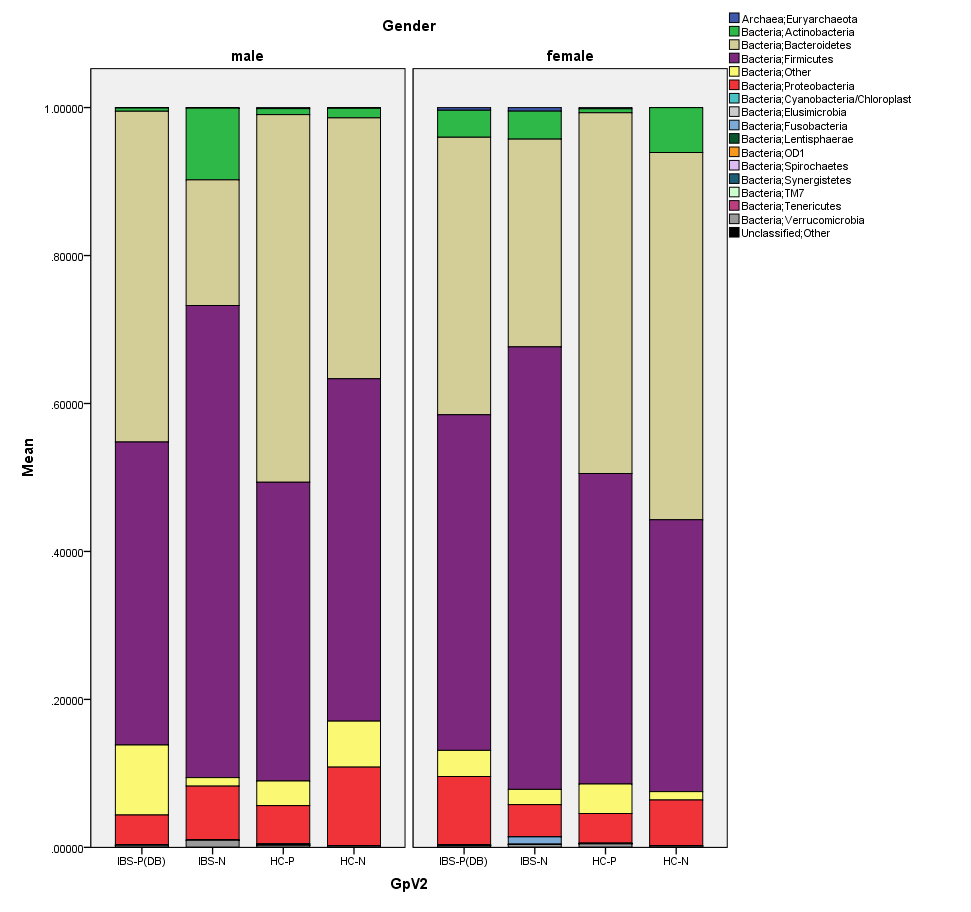

Supplement: Additional file 3: — Clinical subgroup phyla stratified for gender. (DOCX 93 kb) [file 40168_2016_191_MOESM3_ESM.docx]
